# Middle-Aged Worker Bees Express Higher Innate Immunity than Young Worker Bees in the Abdomen without the Digestive Tract of Worker Bees Reared in an Incubator

**DOI:** 10.3390/insects13020209

**Published:** 2022-02-18

**Authors:** Yi-Wen Lin, Chia-Hsiang Chen, Chin-Yuan Hsu

**Affiliations:** 1 Graduate Institute of Biomedical Sciences, College of Medicine, Chang Gung University, Tao-Yuan 333, Taiwan; kiki2208g@gmail.com (Y.-W.L.); cchen3801@gmail.com (C.-H.C.); 2 Department of Biomedical Sciences, College of Medicine, Chang Gung University, Tao-Yuan 333, Taiwan; 3 Department of Psychiatry, Linkou Chang Gung Memorial Hospital, Tao-Yuan 333, Taiwan

**Keywords:** immunity, age, abdomen, digestive tract, honey bee

## Abstract

**Simple Summary:**

Middle-aged worker bees express higher innate immunity than young worker bees in the whole body of worker bees reared in field hives, the whole body of worker bees reared in a 34 °C incubator, and the abdomen without the digestive tract of worker bees reared in a 34 °C incubator. Worker bees raised in an incubator avoid the infection of pathogens and parasites in field hives. The abdomen without the digestive tract is a simplified sample, preventing RNA from the head, thorax, and digestive tract. The abdomen without the digestive tract of worker bees reared in an incubator can be used in studying the relationship between immunity, aging and longevity.

**Abstract:**

Honey bees (*Apis mellifera*) can be reared in an incubator to study the mechanisms of aging and longevity; however, whether breeding in an incubator and using the abdomen without the digestive tract influences the expression of immune genes is unclear. In this study, we assayed the immune genes including *abaecin*, *hymenoptaecin*, *defensin-2*, *glucose dehydrogenase*, *phenoloxidase*, and *lysozyme* from the whole body of young and middle-aged worker bees reared in field hives, the whole body of young and middle-aged worker bees reared in a 34 °C incubator, and the abdomen without the digestive tract of young and middle-aged worker bees reared in a 34 °C incubator. The results showed that three groups of middle-aged worker bees have higher immunity than young worker bees. Furthermore, the similarity of immune genes expression in three groups indicated that the abdomen without the digestive tract of honey bees reared in an incubator can be used to study the relationship between immunity and aging and longevity to avoid the interference of pathogens and parasites from field hives.

## 1. Introduction

Honey bees (*Apis mellifera*) have been reared in an incubator to study aging and longevity [1]. The aging and longevity of honey bees seem to be associated with immunity [2,3]. In addition, the immunity of honey bees has been used to evaluate the impact of pathogens and parasites infection [4,5,6] and to study the relationship of age [3,7]. The innate immune system of honey bees includes humoral and cellular immunity [4].

Humoral immunity involves the synthesis of a battery of antimicrobial peptides in response to infection by bacteria, fungi, or parasites [4]. Humoral immunity consists of at least three antimicrobial peptides, including abaecin [8], hymenoptaecin [9], and defensin [10]. Abaecin, hymenoptaecin, and defensin are produced by adipocytes of the fat body and hemocytes of hemolymph and secreted into the hemolymph [11]. Abaecin was identified from the hemolymph of honey bees after bacterial infection, and it acted against Gram-positive and Gram-negative bacteria [8], and was used to evaluate the antibacterial immune competence of honey bees in different life stages and environmental risks [5]. Hymenoptaecin is a small positively-charged peptide targeting the negatively charged membranes to kill Gram-positive and Gram-negative bacteria [9]. Hymenoptaecin was used to study the immunity of feral and managed honey bee colonies [12] and the evolution of honey bees [13]. Defensins are small antimicrobial peptides that act mainly against Gram-negative bacteria [14]. Defensin-1 is synthesized in salivary glands, and defensin-2 is synthesized in the fat body and lymph [11]. Defensins were used to assess the impact of *Metarhizium anisopliae* infection, lipopolysaccharide, and peptidoglycan on the immunity of honey bees [15,16]. The genes and proteins of abaecin, defensin-2, and hymenoptaecin are used to evaluate the humoral immunity of honey bees [3,4,5].

Cellular immunity involves phagocytosis, nodulation, encapsulation, and melanization [4,17]. Glucose dehydrogenase catalyzes the encapsulation reaction and the killing response to fungal invaders [18]. It was used to study the influence of ectoparasites, such as varroa mites (*Varroa destructor*) on the health of honey bees [19]. Phenoloxidase is a hemolymph protein that mediates nodulation, encapsulation, and melanization [20]. It was used to evaluate the immunity of worker bees, queen bees, and drones with aging [7]. Lysozyme hydrolyzes β-(1,4)-glycosidic bonds of peptidoglycan to eliminate Gram-positive and Gram-negative bacteria [19,21,22] and promotes the expression of other antimicrobial peptides [23]. Lysozyme was used to investigate the impact of microsporidia, such as *Nosema ceranae* on the health of honey bees [4]. The genes of glucose dehydrogenase, phenoloxidase, and lysozyme are used to evaluate the cellular immunity of honey bees [4,18]. 

The immune genes from the whole body [4] and abdomens [3,24] are used to evaluate worker bees’ immunity. In addition, honey bees can be reared in an incubator for aging or longevity studies [1]. Whether breeding in an incubator and using the abdomen without the digestive tract influences the expression of immune genes, the immune genes from the whole body of young and middle-aged worker bees reared in field hives, the whole body of young and middle-aged worker bees reared in a 34 °C incubator, and the abdomen without the digestive tract of young and middle-aged worker bees reared in a 34 °C incubator were assayed to demonstrate that the abdomen without the digestive tract of honey bees reared in an incubator can be used to study the relationship between immunity and aging and longevity.

## 2. Materials and Methods

### 2.1. Honey Bees (Apis mellifera)

The brood combs containing pupae and a few newly emerged worker bees from different colonies were transferred to an incubator (34 °C, 75% relative humidity) [25]. The newly emerged worker bees from brood combs were randomly collected, labeled with white paint, and put into field hives [26]. In addition, the newly emerged worker bees from brood combs were collected in different cages (15 cm × 10 cm × 12 cm), put into a 34 °C incubator (NK system, Yaizu, Shizuoka, Japan), and daily fed with honey and fresh pollen grains mixed with honey (3:1) [1]. The labeled and caged worker bees were collected on the 5th days and 25th days from field hives and cages, respectively. Fifth day-collected worker bees were used as young worker bees, and 25th day-collected worker bees were used as middle-aged worker bees. Young and middle-aged worker bees were collected for the same experiments.

### 2.2. RNA Isolation and Quantitative Real-Time Polymerase Chain Reaction (qPCR) Analysis

5-day-old or 25-day-old worker bees were collected and anesthetized on ice. Total RNA was extracted from the whole body of individual 5-day-old or individual 25-day-old worker bees reared in field hives, the whole body of individual 5-day-old or individual 25-day-old worker bees reared in a 34 °C incubator, and the abdomen without the digestive tract of individual 5-day-old or individual 25-day-old worker bees reared in a 34 °C incubator using Trizol^®^ Reagent (15596018; Invitrogen, Carlsbad, CA, USA) [25]. To prepare the abdomen without the digestive tract, the digestive tract was pulled out from the anus of the abdomen by tweezers. The abdomen without the digestive tract contains the cells of the fat body and hemolymph under the diaphragm [27]. RNA concentration and quality were determined using a Synergy^TM^ HT multi-mode microplate reader (7091000; BioTek, Winooski, VT, USA). The complementary DNA (cDNA) synthesis was performed using an iScript™ cDNA synthesis kit (170-8891; Bio-Rad Laboratories, Irvine, CA, USA). Amplification was performed in a TProfessional Thermocycler (070-851; Biometra, Jena, Germany). Each reaction contained 1 μg of total RNA in a 20 µL reaction volume. The qPCR was performed using a CFX connect RT-PCR detection system (Bio-Rad Laboratories), and each reaction contained 0.5 µL of 10 µM of each primer, 12.5 µL of SYBR Green (170-8882; Bio-Rad Laboratories), 1 µL of diluted cDNA, and 10.5 µL of ddH_2_O in a final volume of 25 μL. The *β-actin* gene was used as a reference gene when measuring gene expression in honey bees [16,25,28,29,30]. The primers were designed according to GenBank’s nucleotide sequences, and primer sequences are shown in Table 1. The PCR program was 95 °C for 3 min, followed by 39 cycles of denaturation at 95 °C for 10 s and annealing at 60 °C for 30 s [25]. All samples were run in quadruplicate [25]. The relative expression levels of genes were calculated using the 2^−ΔΔCt^ method [31]. This experiment was performed with ten biological replicates using a total of ten young and ten middle-aged worker bees.

### 2.3. Statistical Analysis 

Differences in the mean values between the two age groups of bees were examined using two-sample *t*-tests. A *p*-value of less than 0.05 was considered significant.

## 3. Results

### 3.1. Immune Genes Expression in the Whole Body of Worker Bees Reared in Field Hives

**Figure 1:**
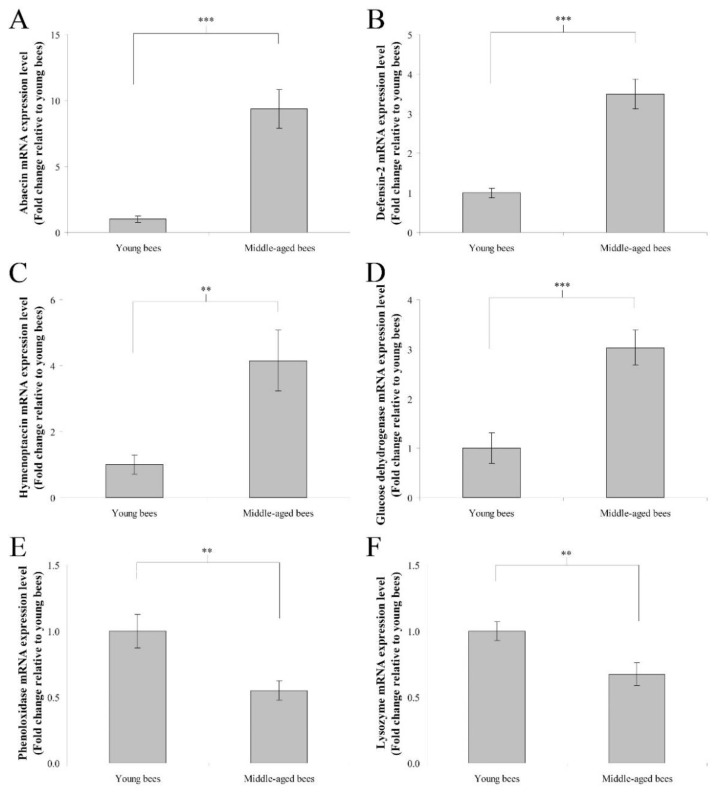
The genes expression of abaecin, defensin-2, hymenoptaecin, glucose dehydrogenase, phenoloxidase, and lysozyme from the whole body of young and middle-aged worker bees reared in field hives. The mRNA expression levels of abaecin (**A**), defensin-2 (**B**), hymenoptaecin (**C**), glucose dehydrogenase (**D**), phenoloxidase (**E**), and lysozyme (**F**) genes were normalized to young worker bees and shown as fold changes, representing the mean ± standard error of the means (SEMs) (*n* = 10). The asterisks indicate significant differences (** *p* < 0.01, *** *p* < 0.001; two-sample *t*-test).

The mRNA expression levels of immune genes including abaecin, hymenoptaecin, defensin-2, glucose dehydrogenase, phenoloxidase, and lysozyme were assayed to determine genes expression in the whole body of worker bees reared in field hives. In humoral immunity, the fold change in the mean abaecin mRNA expression level of middle-aged worker bees was 9.35 ± 1.47 compared with young worker bees (*n* = 10, *p* < 0.001; Figure 1A). The fold change in the mean defensin-2 mRNA expression level of middle-aged worker bees was 3.50 ± 0.38 compared with young worker bees (*n* = 10, *p* < 0.001; Figure 1B). The fold change in the mean hymenoptaecin mRNA expression level of middle-aged worker bees was 4.15 ± 0.93 compared with young worker bees (*n* = 10, *p* < 0.01; Figure 1C). In cellular immunity, the fold change in the mean glucose dehydrogenase mRNA expression level of middle-aged worker bees was 3.03 ± 0.35 compared with young worker bees (*n* = 10, *p* < 0.001; Figure 1D). The fold change in the mean phenoloxidase mRNA expression level of middle-aged worker bees was 0.55 ± 0.07 compared with young worker bees (*n* = 10, *p* < 0.01; Figure 1E). The fold change in the mean lysozyme mRNA expression level of middle-aged worker bees was 0.67 ± 0.09 compared with young worker bees (*n* = 10, *p* < 0.01; Figure 1F). These results indicated that middle-aged worker bees expressed higher levels of abaecin, defensin-2, hymenoptaecin, and glucose dehydrogenase genes, as well as lower levels of phenoloxidase and lysozyme genes than young worker bees.

### 3.2. Immune Genes Expression in the Whole Body of Worker Bees Reared in a 34 °C Incubator

**Figure 2:**
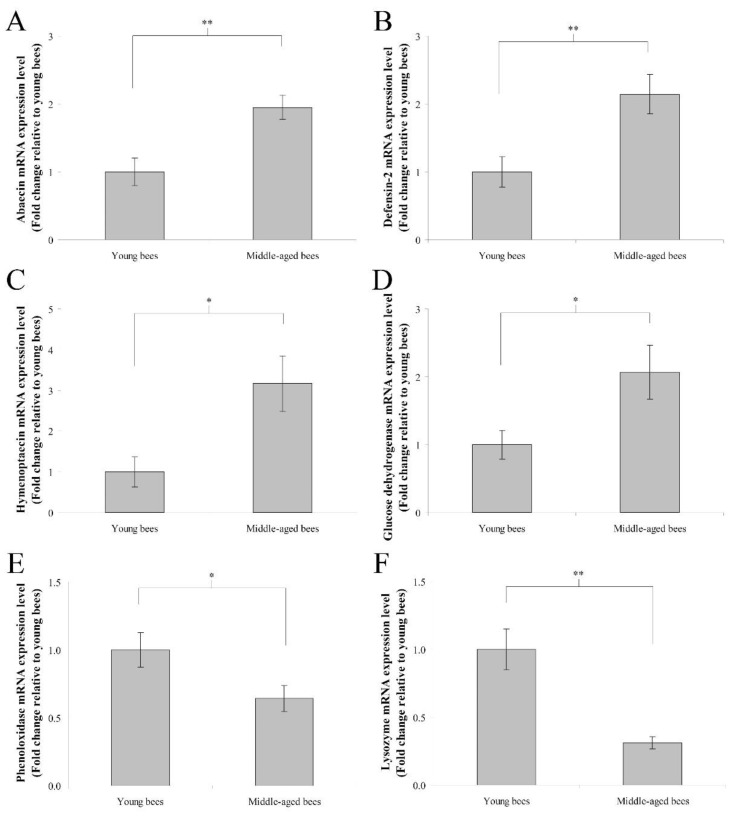
The genes expression of abaecin, defensin-2, hymenoptaecin, glucose dehydrogenase, phenoloxidase, and lysozyme from the whole body of young and middle-aged worker bees reared in an incubator. The mRNA expression levels of abaecin (**A**), defensin-2 (**B**), hymenoptaecin (**C**), glucose dehydrogenase (**D**), phenoloxidase (**E**), and lysozyme (**F**) genes were normalized to young worker bees and shown as fold changes, representing the mean ± SEMs (*n* = 10). The asterisks indicate significant differences (* *p* < 0.05, ** *p* < 0.01; two-sample *t*-test).

The mRNA expression levels of immune genes including abaecin, hymenoptaecin, defensin-2, glucose dehydrogenase, phenoloxidase, and lysozyme were assayed to determine immune genes expression in the whole body of worker bees reared in a 34 °C incubator. In humoral immunity, the fold change in the mean abaecin mRNA expression level of middle-aged worker bees was 1.95 ± 0.18 compared with young worker bees (*n* = 10, *p* < 0.01; Figure 2A). The fold change in the mean defensin-2 mRNA expression level of middle-aged worker bees was 2.15 ± 0.29 compared with young worker bees (*n* = 10, *p* < 0.01; Figure 2B). The fold change in the mean hymenoptaecin mRNA expression level of middle-aged worker bees was 3.17 ± 0.69 compared with young worker bees (*n* = 10, *p* < 0.05; Figure 2C). In cellular immunity, the fold change in the mean glucose dehydrogenase mRNA expression level of middle-aged worker bees was 2.07 ± 0.40 compared with young worker bees (*n* = 10, *p* < 0.05; Figure 2D). The fold change in the mean phenoloxidase mRNA expression level of middle-aged worker bees was 0.64 ± 0.10 compared with young worker bees (*n* = 10, *p* < 0.05; Figure 2E). The fold change in the mean lysozyme mRNA expression level of middle-aged worker bees was 0.31 ± 0.05 compared with young worker bees (*n* = 10, *p* < 0.01; Figure 2F). These results indicated that middle-aged worker bees expressed higher levels of abaecin, defensin-2, hymenoptaecin, and glucose dehydrogenase genes as well as lower levels of phenoloxidase and lysozyme genes than young worker bees.

### 3.3. Immune Genes Expression in the Abdomen without the Digestive Tract of Worker Bees Reared in a 34 °C Incubator

**Figure 3:**
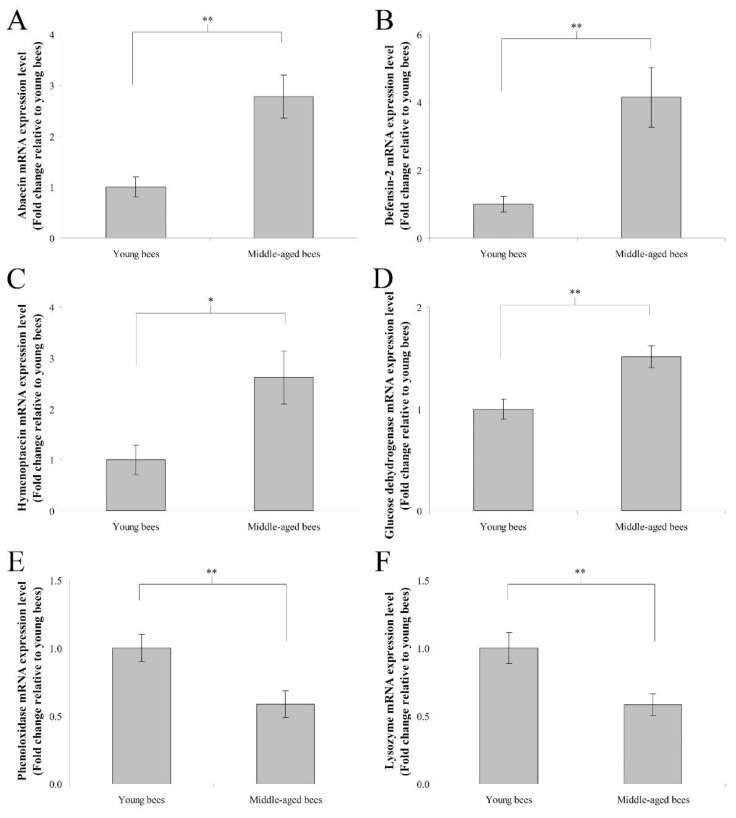
The genes expression of abaecin, defensin-2, hymenoptaecin, glucose dehydrogenase, phenoloxidase, and lysozyme from the abdomen without the digestive tract of young and middle-aged worker bees reared in an incubator. The mRNA expression levels of abaecin (**A**), defensin-2 (**B**), and hymenoptaecin (**C**), glucose dehydrogenase (**D**), phenoloxidase (**E**), and lysozyme (**F**) genes were normalized to young worker bees and shown as fold changes, representing the mean ± SEMs (*n* = 10). The asterisks indicate significant differences (* *p* < 0.05, ** *p* < 0.01; two-sample *t*-test).

The mRNA expression levels of immune genes including abaecin, hymenoptaecin, defensin-2, glucose dehydrogenase, phenoloxidase, and lysozyme were assayed to determine immune gene expression in the abdomen without the digestive tract of worker bees reared in a 34 °C incubator. In humoral immunity, the fold change in the mean abaecin mRNA expression level of middle-aged worker bees was 2.78 ± 0.42 compared with young worker bees (*n* = 10, *p* < 0.01; Figure 3A). The fold change in the mean defensin-2 mRNA expression level of middle-aged worker bees was 4.14 ± 0.88 compared with young worker bees (*n* = 10, *p* < 0.01; Figure 3B). The fold change in the mean hymenoptaecin mRNA expression level of middle-aged worker bees was 2.61 ± 0.52 compared with young worker bees (*n* = 10, *p* < 0.05; Figure 3C). In cellular immunity, the fold change in the mean glucose dehydrogenase mRNA expression level of middle-aged worker bees was 1.51 ± 0.11 compared with young worker bees (*n* = 10, *p* < 0.01; Figure 3D). The fold change in the mean phenoloxidase mRNA expression level of middle-aged worker bees was 0.59 ± 0.10 compared with young worker bees (*n* = 10, *p* < 0.01; Figure 3E). The fold change in the mean lysozyme mRNA expression level of middle-aged worker bees was 0.58 ± 0.08 compared with young worker bees (*n* = 10, *p* < 0.01; Figure 3F). These results indicated that middle-aged worker bees expressed higher levels of abaecin, defensin-2, hymenoptaecin, and glucose dehydrogenase genes, as well as lower levels of phenoloxidase and lysozyme genes than young worker bees.

## 4. Discussion

Immune genes expression from the whole body of young and middle-aged worker bees reared in field hives, the whole body of young and middle-aged worker bees reared in a 34 °C incubator, and the abdomen without the digestive tract of young and middle-aged worker bees reared in a 34 °C incubator were assayed. All three groups showed that middle-aged worker bees exhibited higher innate immunity than young worker bees, indicating that measuring immunity could use three groups. However, worker bees reared in an incubator prevent the infection of pathogens and parasites in field hives. In addition, the abdomen without the digestive tract is a simplified sample that avoids RNA from the head, thorax, and digestive tract. Therefore, the abdomen without the digestive tract of worker bees reared in an incubator can be used in studying the relationship between immunity and aging and longevity.

### 4.1. Middle-Aged Worker Bees Have Higher Innate Immunity Than Young Worker Bees

The mRNA expression levels of abaecin, defensin-2, hymenoptaecin, and glucose dehydrogenase genes in three groups were higher in middle-aged worker bees than in young worker bees, suggesting that the innate immunity of middle-aged worker bees may be higher than that of young worker bees. These results are consistent with a previous study showing that older long-lived winter honey bees increase the gene expression levels of apidaecin-1, defensin-1, and hymenoptaecin [3]. In addition, these findings are supported by previous studies showing that honey bees infected with *Nosema apis* increase the gene expression levels of abaecin, hymenoptaecin, and defensin [4], as well as studies showing that honey bees infected with *E. coli* increase the gene expression levels of abaecin, hymenoptaecin, defensin, and glucose dehydrogenase [19]. Fruit flies also have a similar phenomenon showing that the bacterial load of older fruit flies was significantly lower than that of younger fruit flies, inferred that older flies had better immunity than younger flies [32]. However, the mRNA expression levels of phenoloxidase and lysozyme genes in three groups were lower in middle-aged worker bees than young worker bees. This phenomenon indicated that the higher the immunity, the lower the gene expression levels of phenoloxidase and lysozyme. This inference is supported by previous studies showing that the phenoloxidase activity between nurses and foragers is not significantly different [7] and that honey bees infected with *E. coli* reduce the gene expression levels of phenoloxidase and lysozyme [19]. These results indicated that middle-aged worker bees have higher innate immunity than young workers because there are no pathogens and parasites in an incubator to induce immunity.

The immunity of middle-aged worker bees was higher than that of young worker bees in three groups, but the mRNA expression levels of immune genes of worker bees in field hives were slightly higher than that of those in an incubator. The most likely reason is that worker bees reared in field hives are more susceptible to pathogens and parasites than worker bees reared in an incubator, which leads to increased immunity. This inference is supported by a previous study indicating that the increase in immune gene expression leads to an increased immune response [19].

Previous studies showed that antimicrobial peptides increased with age in *Drosophila* [33,34,35,36,37] and long-lived winter honey bees [3], which infer that older individuals have higher infection rates [2]. In this study, worker bees were reared in an incubator, which is cleaner than field hives, indicating that middle-aged worker bees have higher innate immunity than young worker bees. The inference of high infection rates may be explained by high innate immunity because the high immunity of older worker bees reared in field hives is difficult to distinguish from innate immunity or immunity caused by infection. 

A previous study indicated that young house bees were more susceptible to infection than older forager bees, infected young house bees exhibited higher *abaecin*, *hymenoptaecin*, and *defensin-2* than infected older forager bees, and the immunocompetence of older forager bees did not decline compared to young house bees [15]. These phenomena indicated that older forager bees might have higher innate immunity than young house bees resulting in lower induced immunity in older forager bees than in young house bees.

### 4.2. The Abdomen without the Digestive Tract of Worker Bees Reared in an Incubator Can Be Used to Study the Immunity of Honey Bees

The whole body [4], or abdomens [3,24] were used to extract the mRNA of immune genes for evaluating immunity. The immune gene expression in the whole body of young and middle-aged worker bees reared in field hives, the whole body of young and middle-aged worker bees reared in a 34 °C incubator, and the abdomen without the digestive tract of young and middle-aged worker bees reared in a 34 °C incubator is similar. This phenomenon indicated that the whole body, abdomen, and abdomen with the digestive tract could be used to evaluate immunity. However, worker bees that are reared in an incubator, a cleaner environment can avoid the infection of pathogens and parasites found in field hives. Additionally, the abdomen without the digestive tract avoids RNA from the head, thorax, and digestive tract. Instead, it contains hemolymph and hemolymph cells, such as fat body and hemocytes under the diaphragm [27], keeping immune genes and proteins for assays. Therefore, the abdomen without the digestive tract of worker bees reared in an incubator can be used to study the relationship between immunity and aging and longevity.

## Figures and Tables

**Table 1:** Primer list for qPCR.

Genes	Primer Sequence (5′ → 3′)		Accession Number
*Abaecin*	Forward	CAGCATTCGCATACGTACCA	AF442147.1
	Reverse	GACCAGGAAACGTTGGAAAC	
*Hymenoptaecin*	Forward	CTCTTCTGTGCCGTTGCATA	NM_001011615
	Reverse	GCGTCTCCTGTCATTCCATT	
*Defensin-2*	Forward	GCAACTACCGCCTTTACGTC	NM_001011638.1
	Reverse	GGGTAACGTGCGACGTTTTA	
*GD*	Forward	CTGCACAACCACGTCTCGTT	XM_006567632.1
	Reverse	ACCGCCGAAGAAGATTTGG	
*Phenoloxidase*	Forward	AATCCATTACCTGAAATTGATGCTTAT	NM_001011627
	Reverse	TAATCTTCCAACTAATTCATACGCTCTT	
*Lysozyme*	Forward	ACACGGTTGGTCACTGGTCC	XM_001120136.3
	Reverse	GTCCCACGCTTTGAATCCCT	
*β-actin*	Forward	ATGCCAACACTGTCCTTTCTGG	AB023025.1
	Reverse	GACCCACCAATCCATACGGA	

GD: Glucose dehydrogenase.

## Data Availability

Not applicable.
